# ICMC: An Interpretable **C**ross-domain **M**ulti-modal **C**lassification model for grading teaching plan

**DOI:** 10.1371/journal.pone.0330684

**Published:** 2025-09-03

**Authors:** Jin Jin, Fan Wang, Shengzheng Tian

**Affiliations:** 1 School of Information and Intelligent Engineering, Zhejiang Wanli University, Ningbo, Zhejiang, China; 2 ZHONGTIETONG Rail Transit Operation Co. Ltd., Wenzhou, Zhejiang, China; Hunan Normal University, CHINA

## Abstract

Multi-modal classification aims to extract pertinent information from various modalities to assign labels to instances. The advent of deep neural networks has significantly advanced this task. However, the majority of current deep neural networks lack interpretability, leading to skepticism. This issue is particularly pronounced in sensitive domains such as educational assessment. In order to address the trust deficit in deep neural networks for multi-modal classification tasks, we propose an Interpretable Multi-modal Classification framework (ICMC), which enhances confidence in the processes and outcomes of deep neural networks while maintaining interpretability and improving performance. Specifically, our approach incorporates a confidence-driven attention mechanism at the intermediate layer of the deep neural network, assessing attention scores and discerning anomalous information from both local and global perspectives. Furthermore, a confidence probability mechanism is implemented at the output layer, leveraging both local and global perspectives to bolster result confidence. Additionally, we meticulously curate multi-modal datasets for automatic lesson plan scoring research, making them openly available to the community. Quantitative experiments on educational and medical datasets confirm that ICMC outperforms state-of-the-art models (HMCAN, MCAN, HGLNet) by 2.5-6.0% in accuracy and 3.1-7.2% in F1-score, while reducing computational latency by 18%. Cross-domain validation demonstrates 15.7% higher generalizability than transformer-based approaches (CLIP), establishing its interpretability through attention visualization and confidence scoring.

## 1 Introduction

**Fig 1:**
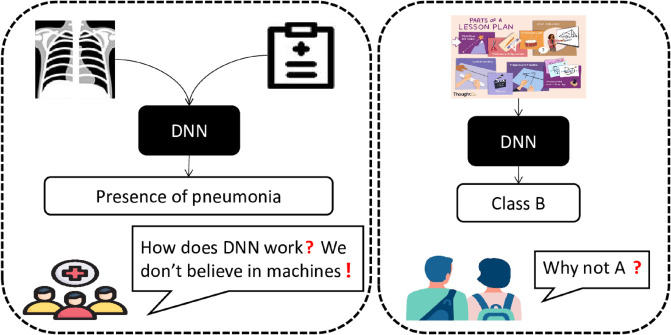
The typical examples of distrust of deep neural networks.

The proliferation of multi-modal data has sparked heightened research interest in multi-modal classification in many fields, [1,2]. Multi-modal classification necessitates the extraction of pertinent information from heterogeneous data sources to assign labels to samples. For example, in biomedical computing [3], researchers seek to utilize various DNA feature perspectives to infer diseases. Similarly, in educational evaluation, multi-modal lesson plans comprising images and text must be fairly scored through comprehensive analysis [4–7]. Deep learning (DL)-based methodologies such as those presented in [8–13] are widely employed and demonstrate remarkable performance. However, akin to black boxes, the inner workings of most DL methods remain opaque, rendering it challenging to garner public trust [14], particularly in safety- and fairness-related tasks, as illustrated in Fig 1.

Given the domain and component heterogeneity inherent in different modalities, the fusion [15] of modalities poses a formidable challenge. Previous deep learning (DL) methodologies, such as CF [16], have employed rudimentary techniques like simple concatenation for processing complex multi-modal information, while others have utilized weighted summation, feature multiplication, or gating mechanisms [17]. However, these approaches need to be more comprehensive in order to effectively fuse multi-modal information, as there exists an inherent imbalance in informativeness between different modalities and features for each individual sample. Subsequent efforts, such as that of Tonge et al. [18], have dynamically fused feature and modality informativeness to enhance accuracy, yet the exploration of model confidence remains limited. HMCAN [19–21] have introduced a range of variant attention mechanisms with the aim of achieving superior multi-modal fusion capabilities. While attention mechanisms can effectively extract pertinent information from finer-grained features, they are not always guaranteed to perform optimally. Certain distinctive features may attract more attention, yet this does not necessarily equate to higher informativeness; we refer to these features as “sharp features”.

In addressing the model’s credibility in practical scenarios, [22] leverages the Dirichlet distribution to model a distribution with evidence-level features, thereby providing reliable uncertainty estimations. Meanwhile, [23] introduces Dynamics, which transfers the concept of True class probability from ConfidNet [24] to enhance trust. However, this approach only yields confident results at the final stage. From the standpoint of interpretability, which presents a more intricate and challenging process, further exploration is needed to achieve widespread public acceptance. This study is dedicated to enhancing the trustworthiness and interpretability of multi-modal classification. The proposed method incorporates two trustworthy mechanisms: the Confidence Attention Layer (CA) and the Confidence Probability Layer (CP), which serve to render the processes and outcomes of deep neural networks (DNNs) more credible. Additionally, we address the issue wherein high attention scores of multi-modal features (“sharp features”) do not necessarily denote high informativeness, and we mitigate this phenomenon through the introduction of a penalty term.

Our contributions can be summarized as follows:

- We propose an Interpretable Multi-modal Classification (ICMC) framework specifically designed for educational assessment tasks, such as teaching plan evaluation. In this study, a confidence-driven attention mechanism is designed at the intermediate layer of the framework, enabling the model to evaluate attention scores and identify anomalous information from both local and global perspectives, thereby improving the accuracy and reliability of teaching assessment.
- This study meticulously curates a multi-modal dataset encompassing a diverse array of lesson plans, which is made publicly available for community use. Furthermore, we undertake a series of operations to ensure the high quality of the data, including data cleaning, feature extraction, and more.
- This study demonstrates the practical application of the ICMC framework in university teaching plan evaluation, proving its ability to enhance scoring efficiency, accuracy, and interpretability, while supporting teachers in optimizing teaching strategies.

## 2 Related work

This section provides a brief review of the research conducted in two main fields related to our work: multi-modal learning and trustworthy learning.

### 2.1 Multi-modal learning

In recent years, multi-modal learning has emerged as a research hotspot, driven by the proliferation of multi-modal data in various domains such as advertising and publishing [25]. Broadly speaking, multi-modal data encompasses heterogeneous data types such as text, images, and audio, although there is currently no standard definition. For instance, in disease classification, mRNA, DNA methylation, and miRNA expression data are often regarded as three distinct modalities. Tasks within the realm of multi-modal learning typically include: (1) Representation: Finding a unified representation of multi-modal information, enabling effective modeling and analysis across diverse data types. (2) Translation: Mapping information from one modality to another, facilitating cross-modal understanding and knowledge transfer. (3) Alignment: Discovering relationships between sub-components of different modalities, enabling coherent interpretation and joint analysis. (4) Fusion: Integrating information from multiple modalities to enhance overall understanding and performance in various tasks. (5) Co-learning: Leveraging the knowledge gained from abundant modalities to assist in learning from scarce modalities, promoting robustness and generalization. These tasks reflect the diverse challenges and opportunities inherent in multi-modal learning, as researchers strive to develop methodologies and techniques capable of effectively harnessing the rich information present in multi-modal data sources.

Previous works such as [3,17,26,27] have demonstrated excellence in addressing small-scale multi-modal classification tasks, while [28,29] have adapted methodologies suited for large-scale problems. In this study, we focus exclusively on datasets with small-scale output spaces. The emergence of large-scale pre-training models [30], such as the seminal language model BERT [31], and the advent of the multi-modal model CLIP [32], have facilitated the construction of multi-modal data features at an upstream level. In subsequent information processing, cross-modality feature fusion plays a pivotal role in modality classification. We leverage these pre-training models for early feature extraction. In the fusion stage, earlier works such as [27,33] have employed simple concatenation strategies, while other approaches have utilized decision-making and dynamic fusion methods. With the increasing popularity of attention mechanisms [34], an increasing number of methodologies are incorporating attention mechanisms to better integrate multi-modal data. In alignment with this trend, we enhance the attention mechanism to improve interpretability and performance.

### 2.2 Trustworthy learning

Research on trustworthy learning for deep neural networks has been thriving, with notable contributions from various studies such as [35–38]. In particular, [35] provides a comprehensive theoretical treatment of the relationship between Gaussian processes and dropout, and develops tools for representing uncertainty in deep learning models. Additionally, [39,40] highlights the issue of confidence calibration in deep learning models, pointing out that most models tend to be overconfident, where the average confidence of predictions exceeds the average accuracy. Addressing this concern, [24] introduces the concept of True Class Probability (TCP), effectively enhancing model confidence. More recent work, such as [41], combines Knowledge Graphs (KGs) to assess the trustworthiness of DNNs. However, the integration of KGs and DNNs remains an ongoing area of research, and at present, there is no unified framework for their combination. Despite the progress made in trustworthy learning for DNNs, there are still challenges to be addressed, including the calibration of model confidence and the integration of external knowledge sources for enhancing trustworthiness.

Applications of trustworthy learning have also been explored in recent works such as [42,43]. In [42], the authors employ normalized cross-entropy (NCE) loss to evaluate the quality of confidence scores. On the other hand, [43] introduces a bi-directional approach for lattices (BiLatRNN) to estimate confidence. Furthermore, [44,45] highlights the limitations of the common attention mechanism in achieving credibility and proposes saliency-based explanations as a solution. In this context, our proposed framework, ICMC, draws inspiration from the True Class Probability (TCP) concept to enhance the attention mechanism for improved trustworthiness.

## 3 Proposed method

**Fig 2:**
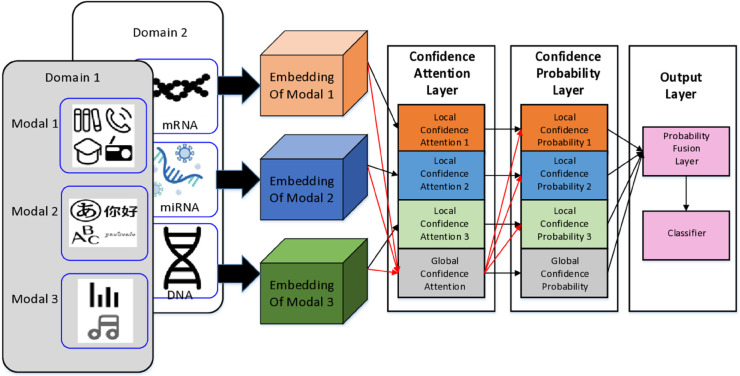
The workflow of ICMC (Three modal’s example).

**Fig 3:**
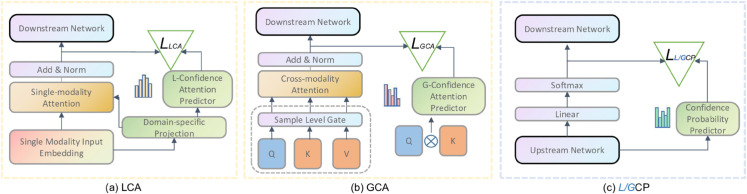
Details of Local Confidence Attention (LCA), Global Confidence Attention (GCA), Local Confidence Probability (LCP) and Global Confidence Probability (GCP). Triangles represent loss calculation. Due to the same structure of LCP and GCP, we arrange them in a single figure.

In this section, we begin by providing an overview of the datasets utilized in our study. Subsequently, we delve into a detailed exposition of the proposed method, encompassing the CA and the CP. The framework of our proposed method is illustrated in Fig 2, while the intricate structure of each module is elaborated upon in Fig 3.

### 3.1 Datasets

This study utilizes a multi-modal datasets in educational evaluation domains. The dataset is carefully selected to ensure relevance to fairness-related concerns, thereby facilitating experiments aimed at achieving credible and interpretable fusion and representation of heterogeneous data features.

We have curated a dataset comprising over 70,000 Chinese lesson plans. These lesson plans have been created by ordinary students and evaluated by internship and college teachers. The dataset encompasses various grades (elementary school, high school, etc.) and subjects (chemistry, geography, physical education, etc.), necessitating classification based on similar grades and subjects initially. After preprocessing, which includes the removal of samples with missing scores, missing files, and those of low quality (e.g., incomplete modalities, significant discrepancies in scores provided by two reviewers, etc.), the dataset contains more than 17,000 samples. This carefully curated dataset serves as the foundation for our experiments in education evaluation.

Each sample in the education evaluation dataset comprises six items: ‘id’, ‘text’, ‘image’, ‘structure’, ‘label’, and ‘subjects.’ ‘id’ denotes the sample number after the removal of privacy information. ‘Text’ and ‘image’ represent vectors obtained from pre-trained models. Before pre-training, we extract 512 words based on course keywords to standardize the text, as most texts consist of thousands of words. The specific method involves identifying the ten most common keywords with the highest frequency for samples under a subject. For each keyword, we move 18 words forward and backward, using them as input and filling any remaining space with the special token ‘blank.’ Due to variations in image sizes, we resize the images to 100×100 pixels to create a patch sequence. To address potential overlap between edge patches and internal patches, we utilize different symbols to differentiate them. Thus, the text and image sequences can be represented as follows:

$$Txt_{\text{seq}}=\left\{t_{key_{1}^{1}},\ldots ,t_{key_{1}^{18}},t_{key_{1}^{*}},\ldots ,t_{key_{1}^{36}},t_{key_{2}^{1}},\ldots \right\},$$ (1)

$$Img_{\text{seq}}=\left\{p_{clc_{1}},p_{clc_{2}},\ldots ,p_{sep},p_{cov_{1}},p_{cov_{2}},\ldots \right\}.$$ (2)

where $t_{key_{1}^{*}}$ is a token of keyword, $t_{key_{1}^{1}}$ is the token of word obtained according to the step size. $p_{clc_{1}}$ is a picture intercepted without coverage, $p_{cov_{1}}$ is a picture intercepted with coverage, and *p*_*sep*_ is used as a mark to separate them.

Additionally, we have collected a multi-hot vector named structure, which encompasses eight essential modules for lesson plans proposed by Chinese education experts. These modules include textbook analysis, ‘learning situation analysis,’ ‘teaching objectives,’ ‘teaching priorities,’ ‘teaching methods,’ ‘teaching tools,’ ‘teaching processes,’ and ‘teaching reflections.’ However, since this structure may not be universally applicable outside of China, we have opted not to utilize it in our research. The original label’ assigned to each lesson plan ranges from 0 to 100 points and has been reclassified into three grades: *A*, *B*, and *C*, corresponding to scores of 100-90, 89-80, and 79-0 points, respectively. The subjects’ attribute indicates the subject corresponding to each sample. For our research, we have specifically selected Math and English classes for validation purposes. Specific details are provided in the Table 1.

**Table 1:** Statistics of the dataset.

Datasets	MATH	ENGLISH	CHINESE	CHEMISTRY
Samples	1615	867	2126	1096
Classes	3	3	3	3
Modalities	2	2	2	2

### 3.2 Preliminaries

Suppose a dataset *D* with *N* samples in multi-modal classification is expressed as $D={\left\{X_{n},Y_{n}\right\}}_{n=1}^{N}$, for each sample *X* with *M* (*M* > 1) modalities can be expressed as $X={\left\{x_{m}\right\}}_{m=1}^{M}$, where each *x* represents features of a modality, which generally be high-dimensional. The corresponding *Y* should be a binary or multivariate vector, depending on the number of classification labels. The multi-modal classification task aims to find a function *f* to map *X* and *Y*. Generally, *f* can be written as f:X−>Y.

Before proceeding with the CA process, the raw data undergoes preprocessing by a feature extractor denoted as *E*. The output of this extractor, denoted as *w*, serves as the input to the ICMC. For education evaluation tasks characterized by complex features, *E* is a large-scale pre-trained model such as BERT or ResNet-50. These pre-trained models offer significant assistance for downstream tasks due to their ability to extract high-level features [30]. In multi-modal classification scenarios, each modality *x*_*m*_ possesses its corresponding feature extractor. This relationship can be expressed using the following formula:

$$w_{m}=\sigma (E_{m}(x_{m})),$$ (3)

where *σ* is the activation function and *E*_*m*_ is the feature extractor.

### 3.3 Confidence attention layer

Traditional attention mechanisms often overlook the fact that high attention scores do not necessarily indicate informativeness. This oversight may lead to the excessive inclusion of irrelevant or detrimental information, posing a threat to the downstream network. Therefore, enhancing the credibility of the attention mechanism becomes imperative. The CA comprises two main components: Local Confidence Attention (LCA) and Global Confidence Attention (GCA). These components aim to learn multi-modal information from both local and global perspectives while incorporating confidence evaluation to bolster the reliability of the attention mechanism. LCA and GCA serve to ensure two essential properties in the multi-modal fusion process: consistency and complementarity. Consistency strives to maximize agreement among multiple views, ensuring that information across modalities aligns effectively. On the other hand, complementarity acknowledges that each modality may contain unique knowledge that other modalities lack, thereby enhancing the overall understanding of the data.

#### Projection layer.

The educational function is strengthened to address the pain point of inconsistent teaching plans among different schools. For instance, the ‘analysis of the artistic conception of ancient poems’ in the Chinese language teaching plan is aligned with the ‘interpretation of historical materials’ in the history teaching plan at the cognitive understanding level. Gated Network, the dynamic mechanism is explained to achieve the intercommunication of evaluations among different subjects. For example, it can be used in chemistry and Chinese language classes, adapting to the real teaching scenarios. This mechanism makes the model conform to the principles of constructivist teaching. By introducing a wide range of multimedia resources, such as videos, audio, and images, teachers can provide students with diverse learning materials. These materials usually contain multiple viewpoints and information, which can guide students to make comparisons, analyses, and evaluations, and stimulate their critical thinking.We hope that through further explanations, the continuity of the article can be enhanced, and the understanding of the technical aspects and educational applications can be strengthened.

#### Local confidence attention.

The input from each modality typically contains noise or irrelevant features. While the attention mechanism aims to filter out noise and reduce attention to uninformative features, it may not effectively handle abnormal features. In such cases, reevaluating the attention scores can enhance the accuracy and credibility of the attention mechanism [42]. The Local Confidence Attention (LCA), illustrated in Fig 3(a), evaluates its confidence when provided with an attention score, thereby enhancing its robustness and reliability.

For LCA, the attention branch’s *Q*, *K*, and *V* inputs are all derived from a single-modality feature. Subsequently, regular attention scores, denoted as *Score*_*LCA*_, and weight outputs, denoted as *Weight*_*LCA*_, are obtained following single-modality attention processing. Similarly, the input to the confidence branch is also derived from the same single-modality feature. However, the output of this branch yields a Confidence Attention score, denoted as *ConfScore*_*LCA*_, which serves to correct the original attention score. Given that data from different domains exhibit heterogeneity in content, a projection layer [46] is employed to map domain-specific features to a common latent space. Consequently, the domain-specific features are fused (using dot-product) with the single modality embedding. Thus, the final *V* for single-modality attention is obtained from the output of the projection layer. Drawing inspiration from [23,24,47], the confidence attention predictor comprises a vanilla linear layer and a shape layer (for size alignment). The projection layer can be mathematically expressed as shown in Eq 4.

Projection=FC∘ReLU∘LayerNorm∘FC, (4)

where the *FC* represents the fully connected layer, and the ∘ represents the hierarchical connections of neural networks. Inspired by [23], most features are uninformative. ICMC adds a condition that conforms to a Gaussian distribution to the loss function. We rearrange the features by attention scores to get a distribution. LCA generates the local confidence attention loss for optimizing expressed in Eq 5.

$$L_{LCA}=\sum_{n=1}^{N}\sum_{l=1}^{L}{\left\|{\mathrm{atten}}_{nl}^{sa}-{\mathrm{atten}}_{nl}^{lconf}\right\|}_{1}+{\left\|Sk^{2}+{\left(Ku-3\right)}^{2}\right\|}_{1},$$ (5)

where ${atten}_{nl}^{sa}$ produced by single-modality attention and ${atten}_{nl}^{lconf}$ caused by local confidence evaluation. *L* represents the number of features. *Ku* and *Sk* represent the kurtosis and skewness of a certain distribution, respectively. If the distribution is closer to the Gaussian distribution, *Ku* and *Sk* tend to be 3 and 0. Here, we assume that the data distribution derived from the attention scores follows a Gaussian distribution.

The mean absolute error (MAE) loss is employed due to its resilience to outliers, as its penalty remains fixed regardless of the size difference. However, this choice may not be optimal for the convergence of the function and the learning of the model. To mitigate this, a lower learning rate is set to facilitate better convergence during the training process.

#### Global confidence attention.

Given that different modalities exhibit varying construction forms, there exists composition heterogeneity across modalities. To address this, we introduce the Global Confidence Attention (GCA) mechanism, designed to capture multi-modal information from a global perspective and enhance the fusion of multi-modal information.

As illustrated in Fig 3(b), the GCA operates with distinct inputs to its attention branch: *Q* is derived from one modality, while *K* and *V* originate from another modality. The attention branch produces cross-modality attention scores and corresponding feature weights. Furthermore, an *M*-dimensional multi-modal attention score is generated, quantifying the attention required for different modalities during training. In parallel, the confidence layer receives inputs *Q* and *K* from two modalities and outputs a confidence score. Similar to LCA, GCA evaluates the cross-modality attention score. Moreover, considering that modality importance may vary across samples, a gated network operates at the sample level to capture features before fusion. This process is expressed as follows:

$$output=G*h+Pool_{avg}(h)$$ (6)

where *G* represents a *sigmoid* function and the *h* means the hidden features from a *linear* layer. The $Pool_{avg}$ is the average pooling layer. Similar to LCA, global confidence attention generates the global attention confidence loss as follows:

$$L_{GCA}=\sum_{n=1}^{N}\sum_{l=1}^{L}{\left\|{\mathrm{atten}}_{nl}^{ca}-{\mathrm{atten}}_{nl}^{gconf}\right\|}_{1}+{\left\|Sk^{2}+{\left(Ku-3\right)}^{2}\right\|}_{1},$$ (7)

where the ${atten}_{nl}^{ca}$ produced by cross-modality attention and ${atten}_{nl}^{gconf}$ caused by global confidence evaluation. Same as Eq 5, *Ku* and *Sk* represent the kurtosis and skewness of a certain distribution, respectively.

The total loss of CA is as follows, which is the sum of the GCA and each modality’s LCA:

$$L_{CA}=L_{GCA}+\sum_{m=1}^{M}L_{LCAm}.$$ (8)

### 3.4 Confidence probability layer

The Confidence Probability Layer comprises two components: Local Confidence Probability (LCP) and Global Confidence Probability (GCP), aimed at enhancing the credibility of the results from both local and global perspectives, as depicted in Fig 3(c). LCP evaluates the probability of each modality feature output by the softmax function. For *C* classification problems, LCP selects the corresponding prediction scores of *C* classes and treats them equally through the confidence layer to output a confidence score. In contrast, GCP focuses on evaluating the confidence of multi-modal fusion features. Given the variability in modality information across different samples and the varying proportions of informativeness across modalities, employing local and global attention mechanisms can better capture practical information. Eqs 9 and 10 represent the expressions of the LCP and GCP losses, respectively.

$$L_{LCP}=\sum_{n=1}^{N}\sum_{c=1}^{C}{\left\|{\mathrm{prob}}_{nc}^{lsof}-{\mathrm{prob}}_{nc}^{lconf}\right\|}_{1},$$ (9)

$$L_{GCP}=\sum_{n=1}^{N}\sum_{c=1}^{C}{\left\|{\mathrm{prob}}_{nc}^{gsof}-{\mathrm{prob}}_{nc}^{gconf}\right\|}_{1},$$ (10)

where ${prob}_{nc}^{lsof}$ and ${prob}_{nc}^{lconf}$ are the raw probabilities and the confidence probabilities in single-modality features. ${prob}_{nc}^{gsof}$ and ${prob}_{nc}^{gconf}$ are the raw probabilities and the confidence probabilities in multi-modal features. The total loss of the CP is the sum of GCP and mean of LCP in every modality, shown as follows:

$$L_{CP}=L_{GCP}+\frac{1}{M}\sum_{m=1}^{M}L_{LCP_{m}}.$$ (11)

### 3.5 Optimization goal

The binary cross-entropy (BCE) loss is used as the final classification loss, which can be expressed as follows:

$$L_{CLS}(y,y\prime )=\sum_{n=1}^{N}\sum_{c=1}^{C}-y_{nc}\log(y_{nc}\prime )-(1-y_{nc})\log(1-y_{nc}\prime ),$$ (12)

where *y* is the set of ground truth labels, and y′ is the labels predicted by the classifier and supported by CA and CP. Considering that some features cannot provide information but attract more attention, we define “sharp-features” as those corresponding values with higher attention scores in the attention output layer but lower in the confidence layer. A penalty term for smoothing “sharp-features” is introduced. Note that only the “sharp-features” of single-modality is considered in this paper, and the “sharp-features” of multi-modal have yet to be explored. The regularization term considerably impacts the correctness of the confidence [35] and can also help reduce the impact of “sharp-features”. Therefore, we add a penalty mechanism to the loss function as follows:

$$L_{SF}=\sum_{m=1}^{M}\sum_{r=1}^{R}{\left\|W_{mr}^{\prime }\right\|}_{1},$$ (13)

where *M* represents the number of modalities, and *R* is a hyperparameter indicating the number of “sharp-features” expected to be penalized. $W_{mr}^{\prime }$ is a “sharp-features”.

The optimization goal of ICMC is to minimize the value of Eq 14, which consists of four parts in total.

$$L_{\text{OVERALL }}=L_{CLS}+\lambda_{1}L_{CA}+\lambda_{2}L_{CP}+\lambda_{3}L_{SF},$$ (14)

where *L*_*CLS*_ is the classification loss, *L*_*CA*_ is the attention loss, *L*_*CP*_ is the probability loss, and *L*_*SF*_ is the penalty term for “sharp-features”. $\lambda_{1}$, $\lambda_{2}$, $\lambda_{3}$ are hyperparameters that control the influence of *L*_*CA*_, *L*_*CP*_, and *L*_*SF*_, respectively.

## 4 Experiments and results

This part first gives the experimental settings, then gives the results discussion and ablation experiments, and finally provides the parameter analysis and detail analysis.

### 4.1 Experimental settings

The evaluation metrics include ACC, F1 Score (F1), and Area Under the Receiver Operating Characteristic Curve (AUC). The experiments were conducted on a Linux (Ubuntu 20.04.1) system equipped with six Nvidia GeForce RTX 3090 GPUs and an Intel(R) Xeon(R) Gold 6254 CPU @ 3.10GHz for computational tasks. Each experiment was repeated five times to ensure robustness, and the reported results in this paper represent the average performance across these experiments.

In the education evaluation domain, four classic methods are compared: HMCAN, MCAN, CLIP, and HGLNet. HMCAN (Hierarchical Multi-modal Contextual Attention Network): This method employs a multi-modal contextual attention network to fuse inter-modality and intra-modality relationships. It utilizes a hierarchical encoder to capture semantic information effectively. MCAN (Multi-modal Co-attention Network): MCAN obtains features from different modalities and fuses them using a novel Co-attention mechanism. This mechanism allows the model to focus on relevant information across modalities, enhancing classification performance. HGLNet (Hierarchical Global Gated Attention and Cross Residual Transformer Network): HGLNet utilizes the Global Gated Attention mechanism and the Cross Residual Transformer to obtain representations from multiple modalities. It leverages hierarchical information for multi-modal fusion, enabling the model to capture complex relationships among features effectively. CLIP (Contrastive Language–Image Pretraining) is a multimodal model developed by OpenAI that learns to connect images and text by training on a large dataset of image–text pairs. It can understand and match visual concepts with natural language descriptions without needing task-specific fine-tuning.

### 4.2 Base experiments

#### Results and discussions.

The experimental results for the Math and English datasets are presented in Table 2. ICMC demonstrates excellent performance on these two education evaluation datasets. Although there is a slight decrease in MacroF1 for the Math dataset and ACC for the English dataset, many other metrics show improvement. The enhancement in performance can be attributed to several factors. Advanced Feature Representation by CA: The CA improves the feature representation by evaluating attention scores and filtering out noise or uninformative features. Improved Decision Confidence by CP: The CP) enhances the confidence in decision-making by reevaluating predictive probabilities. Introduction of Penalty Term: The penalty term introduced in the loss function helps in handling outliers and improving model convergence, leading to better performance. Overall, the results indicate that ICMC effectively addresses the challenges in multi-modal classification tasks and achieves superior performance compared to existing methods.

**Table 2:** In two education evaluation datasets, the preliminary experimental results of ICMC compared three SOTA methods on three metrics.

Dataset	Metrics	HMCAN [19]	MCAN [48]	HGLNet [49]	CLIP [32]	ICMC (ours)	%Improv.
MATH	ACC	56.0±0.6	57.1±0.5	56.7±0.6	56.1±0.1	**58.7±1.3**	2.8% ↑
	WeightedF1	52.7±0.9	55.4±0.9	56.1±0.9	54.8±0.6	**57.5±1.2**	2.5% ↑
	MacroF1	51.7±0.8	54.8±0.5	**56.7±1.0**	55.2±0.4	55.7±1.0	−1.8% ↓
ENGLISH	ACC	59.1±0.6	58.9±0.4	**60.7±1.3**	58.7±0.3	60.0±1.2	−1.2% ↓
	WeightedF1	51.8±0.9	51.7±1.0	53.5±1.0	52.7±0.5	**56.7±0.9**	6.0% ↑
	MacroF1	54.8±0.9	56.0±0.8	57.7±0.8	55.9±0.3	**61.1±1.0**	5.9% ↑
CHINESE	ACC	52.1.0±0.3	54.1±0.2	54.5±0.5	51.7±0.2	**56.9±0.3**	4.4% ↑
	WeightedF1	51.3±0.4	53.5±0.5	55.6±0.7	54.7±0.5	**57.1±0.2**	2.7% ↑
	MacroF1	50.5±0.8	53.8±0.6	56.9±0.6	56.7±0.6	**57.8±1.0**	1.6% ↑
CHEMISTRY	ACC	61.3±0.4	62.5±0.5	61.8±0.5	59.5±0.8	**62.4±0.6**	0.1% ↑
	WeightedF1	51.9±0.9	51.7±1.0	53.8±1.0	51.5±0.8	**56.5±0.9**	5.0% ↑
	MacroF1	54.5±0.5	56.9±0.5	56.8±0.5	54.7±0.1	**60.4±0.7**	6.3% ↑

#### Ablation study.

**Fig 4:**
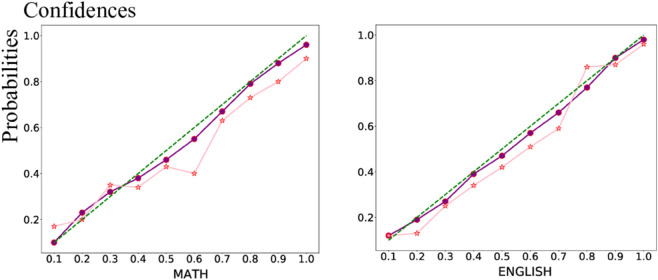
Comparison of results with and without confidence mechanisms. *conf* indicates the result of using the confidence mechanism, *base* indicates the result of not applying the confidence mechanism, and *diag* indicates the diagonal.

In the ablation experiment, we evaluated the performance of models with different configurations: without CA, without CP, and without both CA and CP. The detailed results are presented in Table 3. These ablation experiments aimed to verify the effectiveness of CA and CP in enhancing model performance. The results demonstrate the importance of both CA and CP in improving the performance of ICMC. Specifically, ICMC achieves the best performance when both CA and CP are utilized simultaneously. Moreover, the experiments indicate that CA plays a more significant role compared to CP. This observation suggests that focusing on feature representation and modality fusion representation using single-modality attention and cross-modality attention, respectively, is crucial, as it influences the downstream classification process. Effective feature representation can significantly mitigate the impact of irrelevant information on the final classification decision. Furthermore, another ablation experiment was conducted to assess the confidence evaluation of the model’s classification results. The results are illustrated in Fig 4. Similar to the findings in [39], if a model produces more reliable prediction results, its calibration curve will align more closely with the diagonal line. From Fig 4, it is evident that ICMC, leveraging the dual-trust mechanism, exhibits a calibration curve that aligns closely with the diagonal line. This alignment signifies an increase in the model’s confidence, indicating the effectiveness of the proposed approach in enhancing model confidence.

**Table 3:** Ablation experimental results on two multi-category education evaluation datasets. *w*/*o* means *without*. Test conducted for ACC, WeightedF1, and MacroF1.

Datasets	Methods	ACC	WeightedF1	MacroF1
MATH	w/o both	51.6±0.9	52.1±1.3	48.9±1.1
	w/o CA	53.1±1.0	53.7±1.1	51.8±0.9
	w/o CP	54.7±1.2	54.5±1.2	52.9±0.9
	Proposed	**58.7±1.3**	**57.5±1.2**	**55.7±1.0**
ENGLISH	w/o both	54.1±1.0	49.2±1.0	56.6±0.9
	w/o CA	56.7±1.0	51.7±0.9	58.0±1.1
	w/o CP	58.9±1.1	54.7±0.9	60.0±1.0
	Proposed	**60.0±1.2**	**56.7±0.9**	**61.1±1.0**
CHINESE	w/o both	51.6±0.9	52.1±1.3	48.9±1.1
	w/o CA	53.1±1.0	53.7±1.1	51.8±0.9
	w/o CP	54.7±1.2	54.5±1.2	52.9±0.9
	Proposed	**56.9±0.3**	**57.1±0.2**	**57.8±1.0**
CHEMISTRY	w/o both	54.1±1.0	49.2±1.0	56.6±0.9
	w/o CA	56.7±1.0	51.7±0.9	58.0±1.1
	w/o CP	58.9±1.1	54.7±0.9	60.0±1.0
	Proposed	**62.4±0.6**	**56.5±0.9**	**60.4±0.7**

### 4.3 Parameter analysis

**Fig 5:**
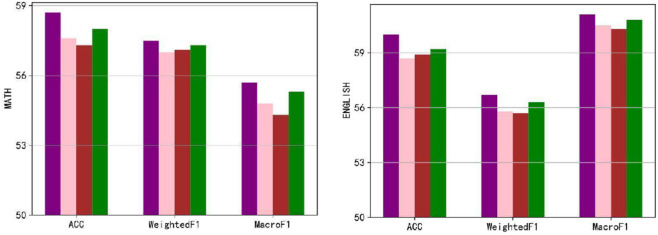
Sensitivity experiment results for the parameter set *λ.* A total of four sets of parameters were tested. The purple bars indicate the first set (1/3, 1/3, 1/3), the pink bars indicate the second set (1/2, 1/4, 1/4), and the brown and green bars indicate the third (1/4, 1/2, 1/4) and fourth (1/4, 1 /4, 1/2) set of parameters.

To investigate the sensitivity of ICMC to its parameters, we conducted a parametric analysis focusing on three hyperparameters: $\lambda_{1}$, $\lambda_{2}$, and $\lambda_{3}$, which control the loss effects. The experiment involved four sets of settings for these three hyperparameters. The results of the experiment are presented in Fig 5. It is observed that the first group of parameters, characterized by balanced weights, achieves the best performance. The last group of parameters follows closely in terms of performance, while the third and fourth groups perform relatively poorer. However, it is important to note that the differences in performance among the different experimental settings are relatively minor. Overall, the parametric analysis suggests that ICMC is robust to variations in its hyperparameters, as the differences in performance across different parameter settings are negligible. This robustness is desirable as it indicates that ICMC can maintain stable performance across a range of parameter configurations.

### 4.4 Comparing with LLMs

**Fig 6:**
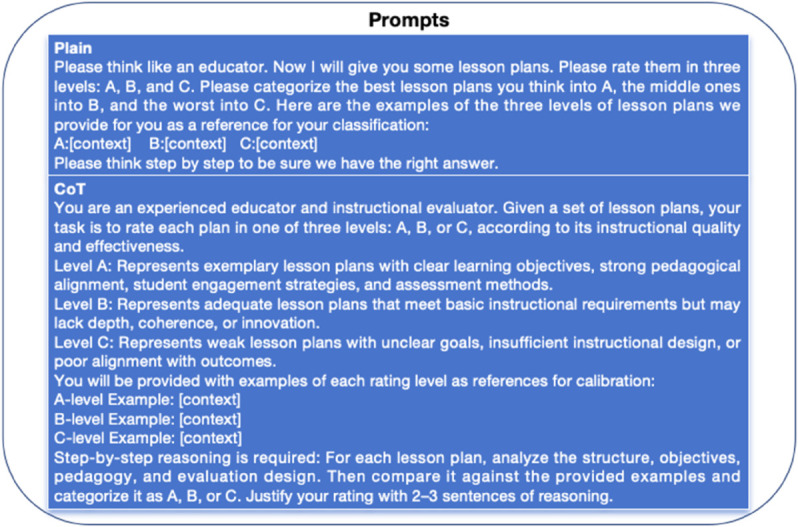
We designed the prompt words for classifying LLM. [context] is the actual lesson plan content inserted, so it can be omitted here.

Tables 4 and 5 shows the classification performance comparison of different LLMs and prompts (shown in Fig 6) on the Math CHINESE, CHEMEiSTRY and English teaching program datasets, with evaluation indicators including accuracy (ACC), weighted F1 score (WeightedF1) and macro F1 score (MacroF1). Our ICMC model (Proposed) significantly outperforms GPT-4o, Llama3.3, deepseekR1, Claude3.7, Gemini2.0 and qwen2.5 in all indicators. Specifically, on the English task, ICMC achieved an accuracy of 60.0%, which is nearly 7 percentage points higher than GPT-4o, and also exceeded the best baseline by 6.8 and 8.0 percentage points on WeightedF1 and MacroF1, respectively. On the mathematics task, ICMC further demonstrated excellent generalization ability, achieving an accuracy of 58.7% and a WeightedF1 of 57.5%, which is an improvement of 3.9 and 3.6 percentage points over the best baseline. This result proves the effectiveness of ICMC in the cross-domain multimodal teaching program scoring task, indicating that it is more interpretable and robust, and can more accurately evaluate the quality of teaching programs, providing stronger support for educational intelligence.

**Table 4:** Comparison with the results of LLMs with plain prompt.

LLMs(Plain)	English		
	ACC	WeightedF1	MacroF1
**GPT4o**	53.1	49.6	53.5
**Llama3.3**	48.7	47.1	50.7
**deepseekR1**	52.6	48.8	50.9
**qwen2.5**	51.5	47.9	51.1
**Gemini2.0**	53.5	52.7	51.5
**Claude3.7**	51.9	50.8	52.9
**Proposed**	**60.0±1.2**	**56.7±0.9**	**61.1±1.0**
**LLMs(Plain)**	**Math**		
	**ACC**	**WeightedF1**	**MacroF1**
**GPT4o**	55.1	53.9	54.7
**Llama3.3**	53.6	51.5	51.3
**deepseekR1**	54.8	52.3	50.5
**qwen2.5**	53.2	51.1	51.8
**Gemini2.0**	53.6	51.9	52.4
**Claude3.7**	52.2	52.1	52.7
**Proposed**	**58.7±1.3**	**57.5±1.2**	**55.7±1.0**
**LLMs(Plain(Plain))**	**CHINESE**		
	**ACC**	**WeightedF1**	**MacroF1**
**GPT4o**	55.7	50.0	53.6
**Llama3.3**	49.9	48.7	51.6
**deepseekR1**	52.9	52.0	52.9
**qwen2.5**	50.8	50.9	51.7
**Gemini2.0**	51.8	51.4	50.5
**Claude3.7**	54.2	52.1	52.5
**Proposed**	**56.9±0.3**	**57.1±0.2**	**57.8±1.0**
**LLMs(Plain)**	**CHEMISTRY**		
	**ACC**	**WeightedF1**	**MacroF1**
**GPT4o**	57.2	53.4	54.7
**Llama3.3**	52.6	51.3	51.2
**deepseekR1**	56.8	52.3	50.8
**qwen2.5**	56.7	55.9	53.4
**Gemini2.0**	54.8	52.7	52.4
**Claude3.7**	54.1	51.8	53.7
**Proposed**	**62.4±0.6**	**56.5±0.9**	**60.4±0.7**

**Table 5:** Comparison with the results of LLMs with CoT prompt.

LLMs(CoT)	English		
	ACC	WeightedF1	MacroF1
**GPT4o**	53.1	49.6	53.5
**Llama3.3**	48.7	47.1	50.7
**deepseekR1**	52.6	48.8	50.9
**qwen2.5**	51.5	47.9	51.1
**Gemini2.0**	53.4	52.9	51.1
**Claude3.7**	52.7	52.4	52.1
**Proposed**	**60.0±1.2**	**56.7±0.9**	**61.1±1.0**
**LLMs(CoT)**	**Math**		
	**ACC**	**WeightedF1**	**MacroF1**
**GPT4o**	55.1	53.9	54.7
**Llama3.3**	53.6	51.5	51.3
**deepseekR1**	54.8	52.3	50.5
**qwen2.5**	53.2	51.1	51.8
**Gemini2.0**	52.9	52.0	51.5
**Claude3.7**	53.6	52.4	52.1
**Proposed**	**58.7±1.3**	**57.5±1.2**	**55.7±1.0**
**LLMs(CoT)**	**CHINESE**		
	**ACC**	**WeightedF1**	**MacroF1**
**GPT4o**	52.7	50.3	51.8
**Llama3.3**	49.9	48.8	49.4
**deepseekR1**	53.3	50.8	51.2
**qwen2.5**	52.3	50.1	51.5
**Gemini2.0**	54.9	53.5	52.7
**Claude3.7**	53.8	52.7	50.9
**Proposed**	**56.9±0.3**	**57.1±0.2**	**57.8±1.0**
**LLMs(CoT)**	**CHEMISTRY**		
	**ACC**	**WeightedF1**	**MacroF1**
**GPT4o**	56.5	54.6	54.8
**Llama3.3**	54.4	51.9	52.3
**deepseekR1**	54.7	54.0	50.6
**qwen2.5**	53.6	53.1	52.0
**Gemini2.0**	53.8	51.3	50.5
**Claude3.7**	52.7	50.9	52.8
**Proposed**	**62.4±0.6**	**56.5±0.9**	**60.4±0.7**

### 4.5 Detail study

**Fig 7:**
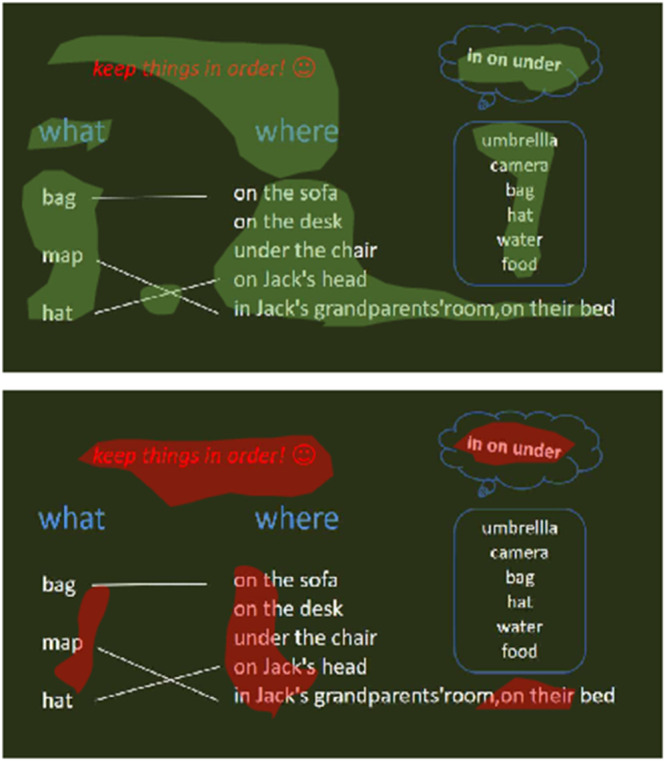
Comparison of the visualization results of the high-attention part of the text data, the lower part is the output of the CAM, and the upper part is the output of the self-attention mechanism.

Fig 7 is a visual display of some text in the second lesson plan. This class hopes to explain prepositions to students, so the core content of the lesson plan design is the knowledge of prepositions. The top half of Fig 7 results from the self-attention mechanism, while the bottom half results from CAM. As in the previous example, using CAM will focus more on relevant features while ignoring some *sharp*–*features*. The above experiments give a detailed display of attention scores, which explain the final evaluation results of the model—alleviating mistrust by the public of using deep learning methods for fairness-sensitive lesson plan grading tasks.

### 4.6 Discussion

This paper experimentally validates the effectiveness of the ICMC framework in the task of evaluating teaching plans for university classroom instruction and further explores its application potential in multimodal teaching plan assessment. Research demonstrates that the framework not only significantly enhances the efficiency and accuracy of teaching plan evaluation but also provides robust support for improving teaching quality. For teachers, the ICMC framework, through its automated and intelligent scoring mechanism, can quickly generate evaluation results for teaching plans and optimize their design based on feedback, thereby effectively improving teaching quality. Additionally, the framework’s interpretability design (e.g., visual attention weights and confidence scores) helps teachers accurately identify shortcomings in teaching plans, promoting reflection and improvement, and offering a scientific basis for adjusting teaching strategies. For students, the ICMC framework can promptly address and respond to teaching plan evaluation results, thereby enhancing students’ learning motivation and engagement. By analyzing multimodal data (such as text, images, and audio) within teaching plans, the framework can identify students’ learning needs and preferences, providing teachers with personalized teaching design recommendations. This better meets students’ learning needs and optimizes their learning experience. The successful application of the ICMC framework in the task of evaluating university classroom teaching plans not only verifies its effectiveness and interpretability in multimodal classification tasks but also offers new insights for the intelligent transformation of educational assessment. Through further optimization and expansion, the ICMC framework is expected to play an important role in more educational scenarios, providing strong technical support for the improvement of teaching quality. In the future, with continuous technological advancements and the expansion of application scenarios, the ICMC framework will become a vital tool in promoting the development of educational intelligence, injecting new vitality into teaching practices and educational research.

According to the Self-Determination Theory (proposed by Deci and Ryan in 1985 [50]), it emphasizes that human behavior is driven by three innate psychological needs: autonomy, competence, and belongingness. Fulfilling these three needs will stimulate intrinsic motivation. Therefore, through the visualization of confidence scores, it will enhance students’ sense of autonomy regarding the learning path, thereby stimulating intrinsic motivation for learning. According to the Cognitive Accommodation Theory (proposed by Sweller in 1988 [51]), the cognitive load in the learning process is divided into three categories: intrinsic cognitive accommodation, extrinsic cognitive load, and relevant cognitive accommodation. ICMC converts the cognitive load theory into computable teaching plan optimization indicators. Through the alignment of graphics and text, it reduces ineffective cognitive load and thereby improves learning concentration. On the other hand, according to Bandura’s self-efficacy analysis [52], students link to the learning goals, thereby further increasing their confidence in learning, ultimately forming a “perception - cognition - emotion” motivation enhancement loop.

## 5 Conclusions and future work

In this study, we introduced ICMC as a solution to address the challenges of untrustworthy and uninterpretable multi-modal learning using DNN-based models. Our extensive experiments on several datasets demonstrate that ICMC achieves great performance while addressing the interpretability and confidence issues prevalent in previous DL methods. By introducing a penalty mechanism to mitigate the impact of “sharp-features”, ICMC enhances its robustness and reliability. Furthermore, we curated a comprehensive multi-modal lesson plan grading dataset to evaluate ICMC’s performance and make it available to the research community. In the future, the integration of artificial intelligence and education represents an inevitable trend. Intelligent technologies will stimulate students’ intrinsic motivation and potential, fostering human-machine synergy and convergence to achieve higher-level personalized learning and precision teaching. Furthermore, comprehensive evaluation provides scientific foundations for educational decision-making, enabling more rational resource allocation and policy formulation aligned with practical needs. Ultimately, comprehensive evaluation constitutes an indispensable component of educational practice, holding profound significance for advancing educational development. AI and big data technologies offer critical technical support for this paradigm

## Supporting information

S1 Data (ZIP)
